# Knockdown of YAP inhibits growth in Hep-2 laryngeal cancer cells via epithelial-mesenchymal transition and the Wnt/β-catenin pathway

**DOI:** 10.1186/s12885-019-5832-9

**Published:** 2019-07-03

**Authors:** Xiaomin Tang, Yuxuan Sun, Ganglun Wan, Jiaqiang Sun, Jingwu Sun, Chunchen Pan

**Affiliations:** Department of Otolaryngology-Head and Neck Surgery, The Provincial Hospital affiliated to Anhui Medical University, Hefei, Anhui People’s Republic of China

**Keywords:** YAP, laryngeal cancer, Wnt/β-catenin, Hep-2, EMT

## Abstract

**Background:**

Yes-associated protein (YAP) plays a crucial role in tumour development and it is the main effector of the Hippo signalling pathway. However, the mechanism underlying YAP downregulation in laryngeal cancer is still unclear. In our previous study, we found that YAP, compared with adjacent tissues, was expressed higher in laryngeal cancer and was also closely associated with histological differentiation, TNM stage and poor prognosis.

**Methods:**

In this study, we attempted to determine whether silenced YAP could downregulate human laryngeal carcinoma Hep-2 cells progression. YAP was downregulated in Hep-2 cells by shRNA, and the malignant ability of Hep-2 was assessed in vitro and in vivo.

**Results:**

In vitro, CCK-8, colony formation and wound healing assays showed that downregulation of YAP significantly reduced the rates of proliferation, migration, and invasion in Hep-2 cells. Downregulation of YAP distinctly induced G2/M cycle arrest and increased the rate of apoptosis. Accordingly, western blot assay suggested that the expression of DKK1, vimentin and β-catenin was significantly decreased after YAP downregulated treatment, thereby indicating that YAP mediated the EMT programme and the Wnt/β-catenin signalling pathway in carcinoma of the larynx. Furthermore, silencing of YAP suppressed Hep-2 cell tumourigenesis and metastasis in vivo.

**Conclusion:**

In summary, our findings demonstrated the proliferation of YAP downregulation and the invasion of Hep-2 cells via downregulating the Wnt/β-catenin pathway in vitro and in vivo, suggesting that YAP may provide a potential therapeutic strategy for the treatment of laryngeal cancer.

## Background

Laryngeal cancer is known as the leading cause of death in otolaryngology worldwide, which accounts for 26–30% of head and neck tumours [1]. Almost 95% of the histological pathology of laryngeal cancer is squamous carcinoma, and the five-year survival rate for patients reaches 64%[2]. However, the incidence of laryngeal cancer has been increasing in recent years. The survival rate of chemotherapy-treated laryngeal cancer have been demonstrated in patients, when compared with traditional surgery [3]. Importantly, gene target therapy is regarded as a significant treatment that has shown better clinical effects for advanced laryngeal cancer with fewer side effects. Until now, we still lack an effective biomarker which affect tumour invasion, proliferation and metastasis in laryngeal cancer patients. Therefore, it is crucial to search for more effective biomarkers and therapeutic targets, which mainly aim to improve the survival of laryngeal cancer patients.

Yes-associated protein (YAP), a promoter of ligand binding, can regulate signal transduction and gene transcription in cells. It also plays a vital role in regulating tissue regeneration and cancer development [4]. Previous studies demonstrated that YAP was highly expressed in most human cancer tissues such as liver cancer [5]. Another previous study also showed that YAP could stimulate breast cancer growth via certain pathways, such as ZEB1-hTERT signaling pathway [6]. Furthermore, the expression of YAP was positively correlated with pathologic grading and clinical stages, suggesting that YAP is involved in the migration and invasion of tumours. In our previous studies [7], we found that the expression of YAP was significantly increased in LSCC and associated with the malignant status of LSCC, indicating that YAP may be a potential prognostic target in laryngeal cancer. Other relevant studies in vitro and in vivo also confirmed that downregulated YAP inhibited EMT on hepatocellular carcinoma cells [8]. Pan et al.[9] and Zygulska et al.[10] also found similar results in lung cancer and other tumours. These results showed that targeted downregulation of YAP could significantly reduce the malignant transformation ability of various tumour cells.

Recent studies [11] reported that epithelial-mesenchymal transition (EMT) was a distinctive feature of tumours, which was evoked during tumour invasion and metastasis. The ability of epithelial tumour cells to invade interstitial organs plays a crucial part in the development of tumours [12].

The Wnt/β-catenin signalling pathway is pivotal in embryonic development signalling while overexpression of the pathway is closely related to most human cancers [13]. It was found that the overexpression of Wnt could lead to malignant transformation of mouse mammary tissue in the earliest observation. Nowadays, the Wnt pathway evolves as a central mechanism in cancer biology [14]. Available data indicated that a loss of Wnt signalling pathway inhibitors by promoter hypermethylation might impacted NSCLC tumourigenesis and prognosis [15]. There have been recent reports about β-catenin in the cell nucleus related to EMT and tumour migration. Additionally, scientists also found that some proteins could regulate the EMT program via the Wnt/β-catenin pathway in cancer, such as ovarian cancer [16]. Overall, Wnt signalling is complex, especially because it produces factors that may impact tumour aggressiveness through interacting with other pathways [17, 18]. It is not clear whether YAP can regulate the Wnt/β-Catenin pathway and induce EMT in human laryngeal cancer Hep-2 cells, thus exploring the regulatory mechanism may provide new insights into the mechanism of YAP-impacted EMT in laryngeal cancer.

## Methods

### Animals

We purchased 36 BALB/c mice (mean body weight 20 g, male, aged 4 weeks,) from the Model Animal Research Center of Nanjing University, China. The animal research was completed with the approval of the Ethics Board of the University of Science and Technology of China. These BALB/c mice were kept in a specific pathogen-free environment, where humidity was maintained in the range of 50–60% and temperature was at 25 °C. Care and uses of animals were as per the principles and guidelines of the Ethics Board of the University of Science and Technology of China. After 3 weeks of respective treatment, all mice in various groups were sacrificed by over dose of diethyl.

### Cell lines

The laryngeal cancer cell line Hep-2 (FH0119) was purchased from the FuHeng Biology Company (Shanghai, China). Cells were cultured in DMEM (Sangon Biotech, Shanghai, China) supplemented with 10% FBS (Gibco, Sydney, Australia), 1% Penicillin-Streptomycin Solution (Sangon Biotech, Shanghai, China) at 37 °C in the atmosphere of 5% CO_2_. Hep-2 cells were passaged every 2 days by using 0.25% trypsin (Sangon Biotech, Shanghai, China).

### Lentiviral vector construction and plasmid DNA transfection

**Fig. 1:**
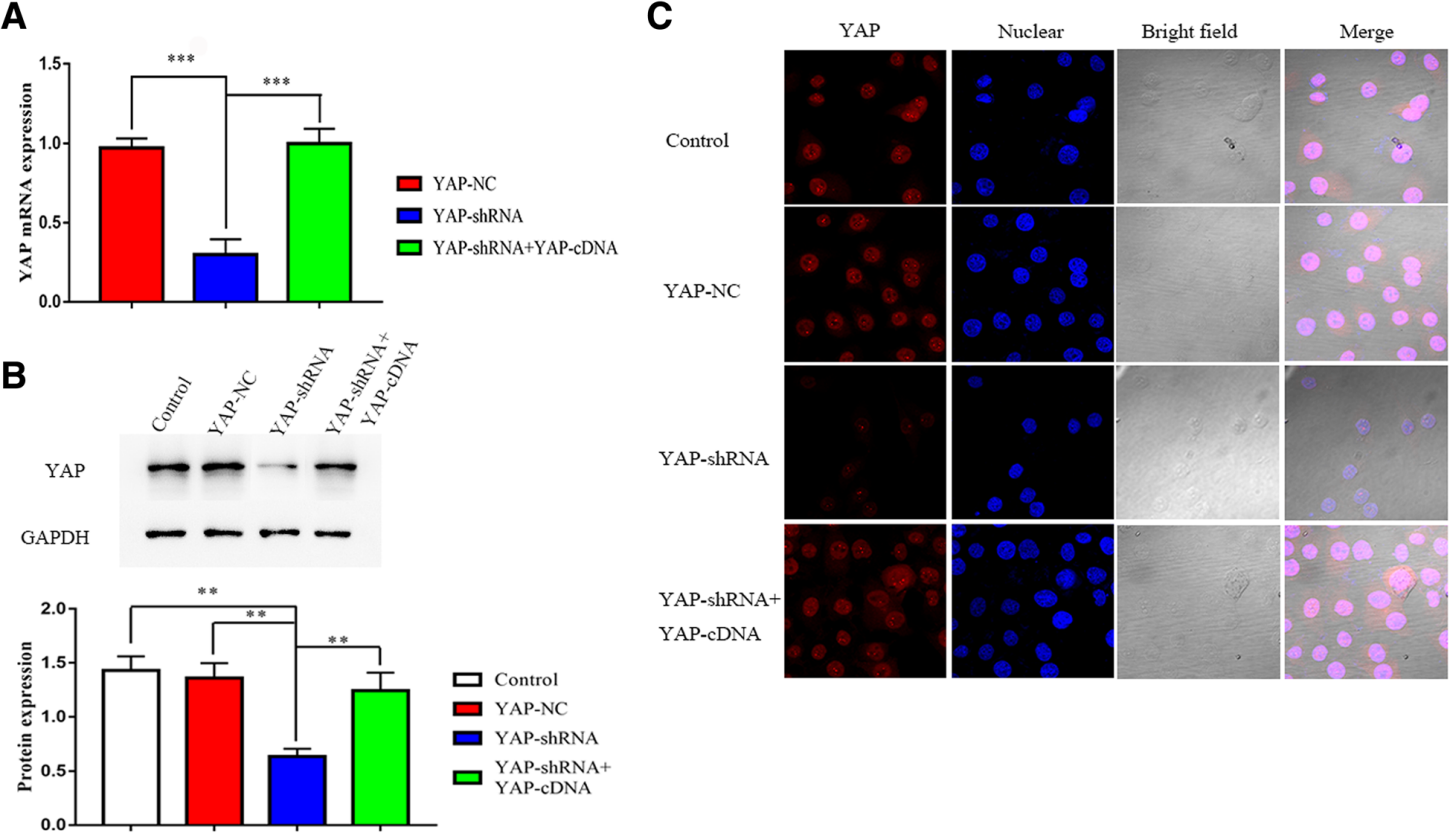
YAP knockdowned significantly decreased YAP expression at mRNA (**a**) and protein (**b**) levels in Hep-2 cells. **c** Immunofluorescence (bottom panel) was used to detect the expression of YAP (red). DAPI (blue) was used to stain the nuclei. The fluorescence intensity of YAP was stronger in YAP-NC groups, and was weaker in cells transfected with shRNA. One representative experiment out of the three performed is shown (80X). Experiments were repeated three times, and data are shown as the mean ± SD, **P < 0.01 (**a**), **P < 0.01 (**b**)

Short hairpin RNA targeting YAP and negative control shRNA were designed and chemically synthesized by the Hanbio Co. Ltd. (Shanghai, China) as shown in Table 1. Hep-2 cells were seeded at a density of 1 × 10^5^ cells/well in twelve-well culture plates one day before transfection. Then cells were transfected by using 0.5 ml polybrene (Hanbio, Shanghai, China) in DMEM culture medium with shRNA according to the manufacturer’s instruction. Cells were incubated at 37 °C in a 5% CO_2_ for 4 h. Next 0.5 ml DMEM culture medium was added and cells were cultured for 24 h. A complete medium containing 2 μg/ml puromycin (Solarbio, Beijing, China) was added, and stably transfected cells were obtained by being filtrated for 2 weeks. Cells were transfected with 4 μg of YAP plasmid DNA by using LipoFiter (Hanbio, Shanghai, China) transfection reagent according to the manufacturer’s protocol. After transfection for 24 h, Hep-2 cells were collected for further researches. The expression of YAP was verified by Western Blot and qRT-PCR assay (Fig. 1).

**Table 1:** shRNA sequences for YAP.

Sequences	
shRNA1	GCTGATGGTTTATGCCACAAGTTGA
Top strand	GATCCGCTGATGGTTTATGCCACAAGTTGATTCAAGAGATCAACTTGTGGCATAAACCATCAGCTTTTTTC
Bottom strand	AATTGAAAAAAGCTGATGGTTTATGCCACAAGTTGATCTCTTGAATCAACTTGTGGCATAAACCATCAGCG
shRNA2	GTCACCGAGATCTTCGGGCTGCTAAT
Top strand	GATCCGTCACCGAGATCTTCGGGCTGCTAATTTCAAGAGAATTAGCAGCCCG AAGATCTCGGTGATTTTTTC
Bottom strand	AATTGAAAAAATCACCGAGATCTTCGGGCTGCTAATTCTCTTGAAATTAGCA GCCCGAAGATCTCGGTGACG
NC-shRNA	TTCTCCGAACGTGTCACGTAA
Top strand	GATCCGTTCTCCGAACGTGTCACGTAATTCAAGAGATTACGTGACACGTTCGGAGAATT TTTTC
Bottom strand	AATTGAAAAAATTCTCCGAACGTGTCACGTAATCTCTTGAATTACGTGACACGTTCGGAG AACG
cDNA	
YES1-F	CGAATTCCCCCGGGCGGGATCCGCCACCATGGGCTGCATTAAAAGTA
YES1-R	CGGCCGCTACGCGTCGGGGTACCTAAATTTTCTCCTGGCTGGTACT

### Cell viability assay in vitro

After stable transfection, cells were digested with 0.25% trypsin for 1 min and counted by a hemocytometer. Cells (2000 cells/well) were seeded into 96-well plates with 100 μl per well of complete medium. Then, cells were incubated at 37 °C in an environment with 5% CO_2_. At the specific time points, before 10 μl CCK-8 (Sangon Biotech, Shanghai, China) solution was added, the medium was replaced by 100 μl fresh culture medium. After incubating 1 h at 37 °C, the absorbance was recorded at 450 nm by using GloMax DISCOVER (Promega, New York, USA). Each sample had three replicates.

### Colony formation assay

One thousand cells of each group were seeded into a 6-well plate with complete medium. The colonies were visible by viewing with the eyes after 7 days. The plates were washed three times in phosphate buffered saline (PBS) after the complete medium was removed. Then the colonies were fixed in Crystal Violet Staining Solution for 15–20 min. Finally, each plate was washed three times with PBS. The experiment was performed three times.

### Wound healing assay

Hep-2 cells were cultured in 6-well cell plates with complete medium until confluent. Wounds were scratched with a 100 μl pipette tip. After the cell debris was washed away by PBS, pictures of each “wound” were taken by using inverted microscope (Olympus, Tokyo, Japan). Cells were incubated in serum-free medium at 37 °C in an environment with 5% CO_2._ At 24 and 48 h, another picture of each “wound” was taken by inverted microscope.

### Immunofluorescence (IF)

Slips in 24-well plates were fixed in 4% paraformaldehyde for 20 min after cells growing on cover it, then the cells were permeabilized with 0.5% Triton X-100 (Solarbio, Beijing, China) for 20 min at room temperature. The cells were incubated at 4 °C overnight with the primary antibody (YAP, 1:100, BOSTER, Wuhan, China) after blocking with normal donkey serum (Solarbio, Beijing, China) for 30 min at 37 °C. Then the cells were incubated for 1 h at 37 °C with TRITC-conjugated donkey anti-rabbit IgG (1200, Sangon Biotech, Shanghai, China) as the secondary antibody. The nuclei were stained by DAPI (Sangon Biotech, Shanghai, China) for 5 min after washed with PBS. Images were captured by using confocal microscope (Olympus, Tokyo, Japan).

### Flow cytometry

Cells (5 × 10^3^ cells/well) were seeded into 6-well plates overnight. An Annexin PE/7-AAD apoptosis detection kit (BD, New York, USA) was used to examine the rate of apoptosis. Data analysis was performed by using Guava easyCyte 12 (Merck Millipore, New York, USA). For cell cycle analysis, Hep-2 cells were fixed in 70% ethanol at 4 °C overnight. Then cells were stained with propidium iodide, and analysed by Guava easyCyte 12 in 1 h(Merck Millipore, New York, USA).

### Quantitative real-time polymerase chain reaction (qRT-PCR) analysis

Total RNA was isolated from cells by using IOSPlus reagent (Takara Bio, Tokyo, Japan). On the basis of the instructions of the reverse transcriptase kit (Takara, Tokyo, Japan), cDNA was synthesized using 2 μg of the total RNA in TProfessional Thermocycler (Biometra, Berlin, German). Then, cDNA samples were subjected to qRT-PCR for 40 cycles by using TB GreenTM Premix Ex TaqTM II (Takara, Tokyo, Japan) in SLAN-96P Real-Time PCR System (HONGSHI, Shanghai, China). Primers (Table 2) were designed with the approval of the Sango Biotech Co. Ltd. (Shanghai, China). GAPDH was used as an internal control. Data of different gene were analyzed using the comparative Ct (2^-ΔΔCt^) method.

**Table 2:** Primer in this study

Gene	Forward	Reverse
YAP	GAACAATGACGACCAATAGCTC	TAGTCCACTGTCTGTACTCTCA
DKK1	TACCAGACCATTGACAACTACC	TCCATTTTTGCAGTAATTCCCG
GSK-3β	AGGAGAACCCAATGTTTCGTAT	ATCCCCTGGAAATATTGGTTGT
β-catenin	TGGATTGATTCGAAATCTTGCC	GAACAAGCAACTGAACTAGTCG
vimentin	ATGTCCACCAGGTCCGTGT	TTCTTGAACTCGGTGTTGATGG
E-cadherin	AGTCACTGACACCAACGATAAT	ATCGTTGTTCACTGGATTTGTG
GAPDH	GTATCGTGGAAGGACTCATGAC	ACCACCTTCTTGATGTCATCAT

### Western blot (WB) analysis

First, total protein was extracted from the cells. Almost 50 μg of crude protein was denatured and electrophoresed on 10% SDS-PAGE gels. Proteins were transferred onto nitrocellulose membranes by electro-blotting after electrophoretic separation, followed by blocking for 1 h at room temperature in PBS with 5% nonfat milk. The blots were incubated with YAP, β-catenin, E-cadherin, vimentin, GSK-3β, DKK1 and GAPDH (information in Table 3) primary antibodies at 4 °C overnight. After wash with PBS-T, membranes were hybridized with appropriate secondary antibody (Santa, New York, USA) at room temperature for 1 h. Last, images of the western blot bands were performed with AlphaEase FC and the intensity in each group was measured with ImageJ analysis software. GAPDH was used as an internal control.

**Table 3:** Antibody in this study

Antibody	Ratio	Art.No.	Brand	Concentration
WB Antibody				
YAP	1:400	BM4253	BOSTER	200μg/ml
DDK1	1:1000	ab61275	Abcam	1 mg/ml
GSK-3β	1:1000	12,456 T	CST	239μg/ml
β-catenin	1:1000	8480 T	CST	239μg/ml
vimentin	1:1000	5741 T	CST	239μg/ml
E-cadherin	1:1000	3195 T	CST	239μg/ml
GAPDH	1:1000	5174 T	CST	239μg/ml
IHC Antibody				
YAP	1:50	BM4253	BOSTER	200μg/ml
Ki67	1:50	BM4381	BOSTER	200μg/ml
BCL-2	1:50	BM0200	BOSTER	200μg/ml

### In vivo tumourigenicity and metastasis assays

Tumour model experiments were performed in BALB/c mice purchased from the Model Animal Research Center of Nanjing University, China. To observe the tumourigenicity, we harvested stably transfected control, YAP-shRNA and YAP-NC cells. First, 18 BALB/c mice were randomly divided into 3 groups (*n* = 6). Then each mouse was subcutaneously inoculated with 1 × 10^6^ cells in the right upper limb to develop xenograft tumours. Sizes of tumours were measured every three days. In order to detect metastasis ability of Hep-2 cells, we randomly divided 18 BALB/c nude mice into 3 groups (n = 6). Then cells of control, YAP-shRNA, and YAP-NC group were injected into the tail vein of BALB/c nude mice. All mice in experiments were sacrificed after three weeks. All the experiments in vivo were performed in specific pathogen-free (SPF) conditions which was provided by the Committee on the Ethics of Animal Experiments of USTC.

### Immunohistochemistry (IHC)

The sections were dewaxed and deparaffinized in xylene and rehydrated in graded alcohol solutions. Then the sections were heated for 30 min in Tris-EDTA buffer by microwave oven. Subsequently, the slides were stained with primary antibodies for YAP, Ki67, and Bcl-2 (information in Table 3) and their respective secondary antibodies. Before dehydration and mounting, the sections were counter-stained with haematoxylin. Images were performed with Olympus microscope camera (DP70, Tokyo, Japan).

### Statistical analyses

The statistical software GraphPad Prism 7.0 was used to analyze significance of data. All data were collected from three or more samples for each of the experimental groups, and are presented as mean values±SEM. Two-tailed Student’s t-tests was used to determine significance when only two groups were compared. *P* values of less than 0.05 was considered statistically significant.

## Results

### Downregulation of YAP by shRNA

Specific silencing YAP by shRNA was applied to conduct loss-of function assays. Untransfected isotype Hep-2 cells (Control group) and negative control shRNA (YAP-NC shRNA) transfected Hep-2 cells were used as controls. QRT-PCR, WB and IF showed that YAP expression were decreased in the cells with small interfering RNA (YAP-shRNA group) (*P* < 0.05; Fig. 1). Subsequently, in YAP rescue experiment, YAP plasmid DNA transfection was retransfected into the cells with YAP-shRNA (YAP-shRNA + YAP-cDNA group), the expression of YAP was significantly elevated (*P* < 0.05; Fig. 1).

### Downregulation of YAP supresses the proliferation, migration, and invasiveness of Hep-2 cells

**Fig. 2:**
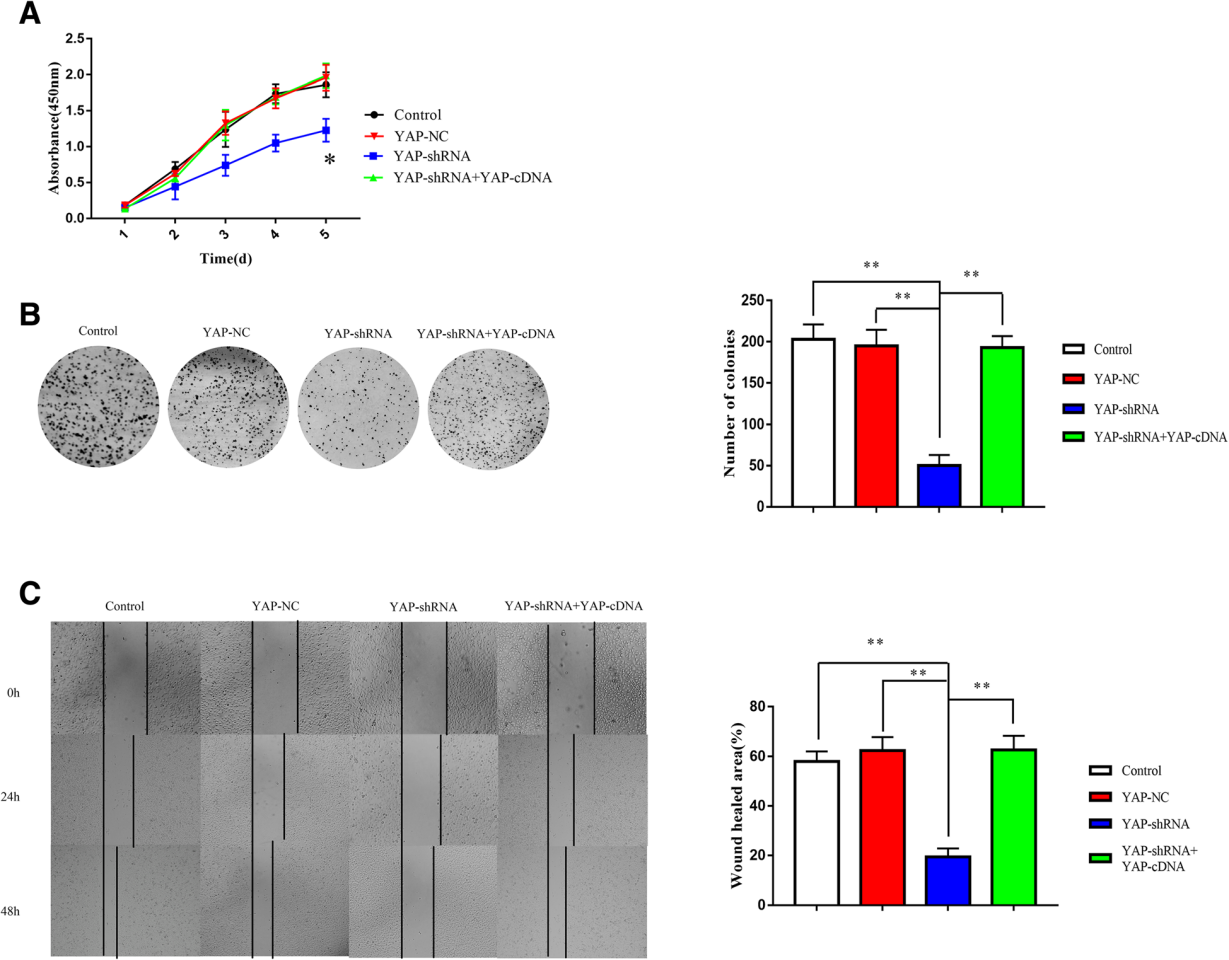
YAP knockdowned suppresses cell proliferation, invasion and migration. Silenced YAP inhibited cell proliferation in Hep-2 cells by CCK-8 (top panel). **a** The OD value at 450 nm for 5 days. Hep-2 with no treatment (Control), Non-nonspecific shRNA treatment (YAP-NC), YAP knockdown (YAP-shRNA) groups, and cDNA overexpression after YAP knockdown (YAP-shRNA+YAP-cDNA), *P < 0.05. **b** Colony formation assays (second panel) were performed to evaluate the proliferative capability of control, YAP-NC, YAP-shRNA and YAP-shRNA+YAP-cDNA cell groups. A representative image is shown, and a statistical comparison of the indicated groups was performed across four independent experiments, **P < 0.01. **c** Wound healing assays (third panel) were performed to explore the migration capability, and solid lines represented the wound edges. Images were captured by using light microscopy (40X). The migration index was calculated as described in the Materials and Methods (control, YAP-NC, YAP-shRNA and YAP-shRNA+YAP-cDNA). Statistical analysis is shown, **P < 0.01

EMT is considered to be a critical step for tumour infiltration and distant metastasis in majority carcinomas. To explore the function of YAP in Hep-2, we designed migration assay, colony formation assay and cell viability assay after stable downregulation of YAP in Hep-2 cells. Comparing the control, YAP-NC, YAP-shRNA and YAP-shRNA+YAP-cDNA groups (Fig. 2a), we found that the YAP-shRNA group had lower cell viability than others. In addition, the wounds in the YAP-shRNA group healed more slowly. However, cell migration was increased after transfection by YAP-cDNA (Fig. 2c). Furthermore, the colony formation of cells decreased in the YAP-shRNA group while the other three groups remained the same (Fig. 2b). These results indicated that downregulated YAP might inhibit growth in Hep-2 cells.

### Downregulation of YAP increases the apoptosis and induces G2/M cell cycle arrest in Hep-2 cells

**Fig 3:**
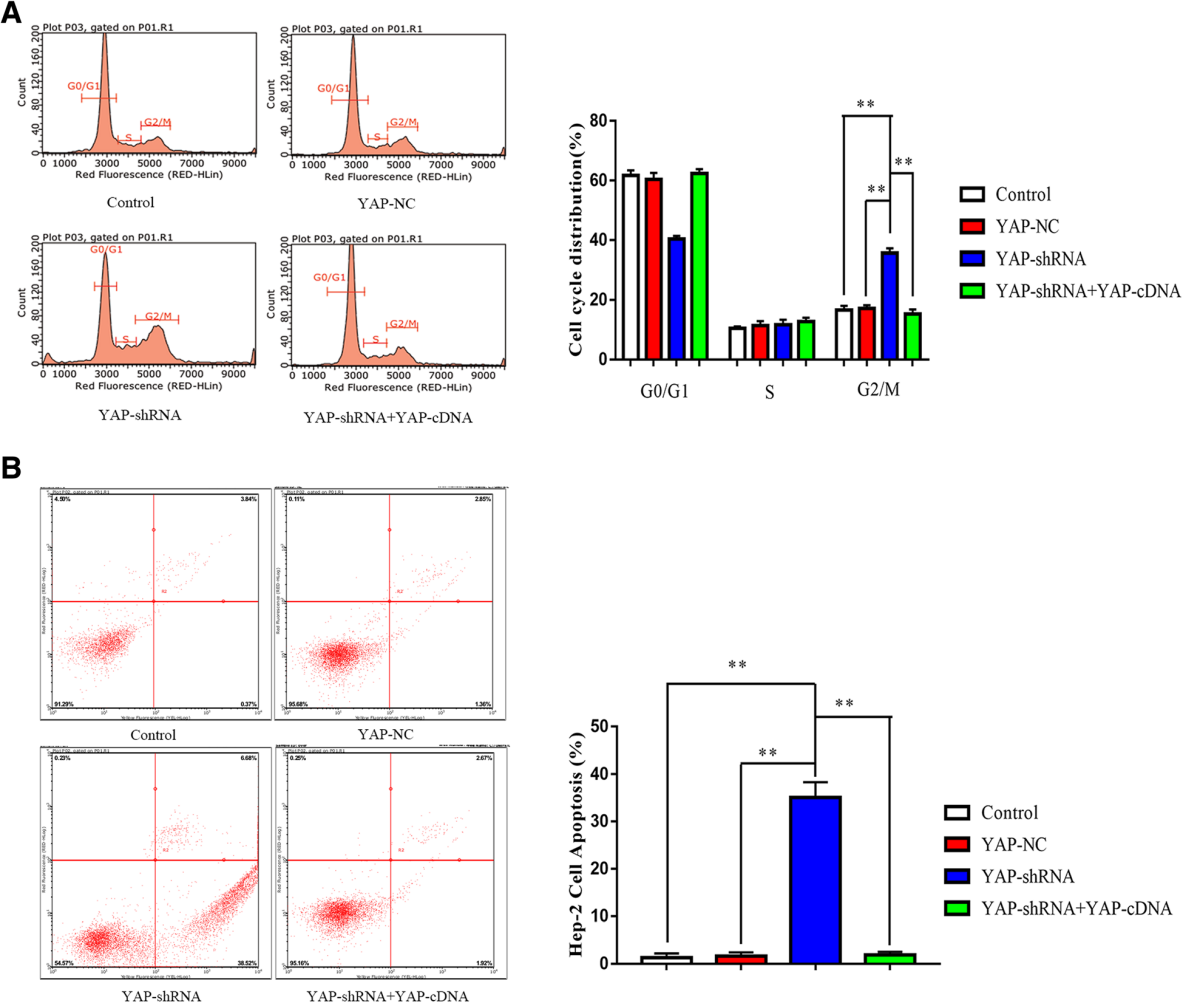
Silencing of YAP promotes cell apoptosis and induces cell cycle arrest. **a** Guava easyCyte12 was used to investigate differences in cell cycle (top panel) distribution following YAP silencing or overexpression in Hep-2. Silenced YAP drove G2/M arrest in control, YAP-NC, YAP-shRNA and YAP-shRNA+YAP-cDNA cell groups. In addition, when YAP knockdown Hep-2 cells overexpressed YAP, the G2/M phase distribution was decreased, **P < 0.01; **b** Apoptotic cells (medium panel) were analysed by Guava easyCyte12 via staining of annexin PE/7-AAD. The percentage of apoptotic cells is shown. **P < 0.01

YAP-downregulated induced G2/M cell cycle arrest in Hep-2 cells (*P* < 0.01, Fig. 3a), and the percentage of Hep-2 was also markedly decreased in G0/G1 phase (*P* < 0.01, Fig. 3a). Additionally, Annexin PE/7-AAD double-staining assays showed that the rate of apoptosis in Hep-2 treated with YAP-downregulated was significantly higher than YAP-NC group, the control group and the YAP-shRNA+YAP-cDNA group (*P* < 0.01, Fig. 3b).

### Downregulation of the YAP reduces mRNA and protein levels by inducing the EMT programme and Wnt/β-catenin pathway

**Fig. 4:**
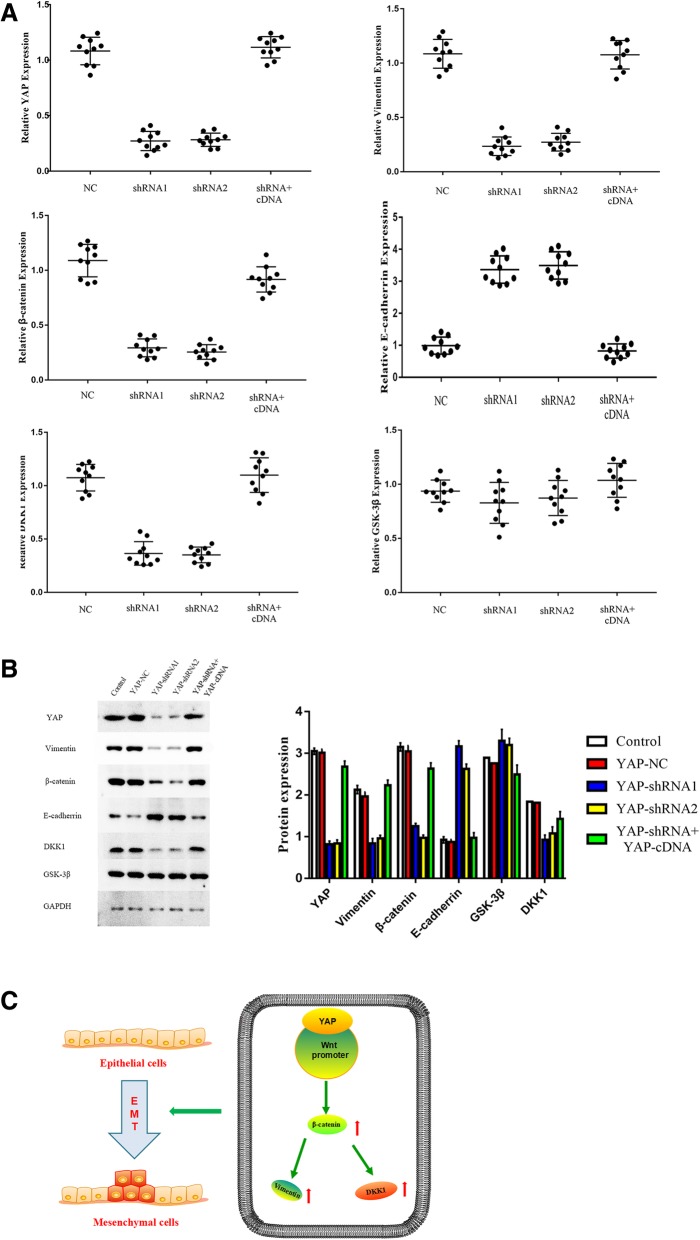
**a** Targeted silencing of YAP inhibits the expression of mRNA and protein levels. **a** qRT-PCR was used to analyse the mRNA expression of YAP, GSK-3β, DDK1, E-cadherin, β-catenin and vimentin in Hep-2 cell lines after transfection or not, and GAPDH was used as the internal control. Experiments were repeated ten times, and data are shown as the mean ± SD, **P < 0.01. **b** Western blot assay was employed to investigate the expression of YAP, β-catenin, E-cadherin, vimentin, DDK1 and GSK-3β. WB results indicated that the expression of vimentin and β-catenin were downregulated with YAP knockdown and were upregulated when cells were administered YAP-cDNA. Representative images are shown. Statistical analysis of the relative optical density of each band is shown. GAPDH was used as an internal control, **P < 0.01. **c** Model of the role of YAP in laryngeal cancer

We analysed the mRNA and protein levels of epithelial markers and mesenchymal markers after inhibition and overexpression of YAP gene in Hep-2 cells to determine whether YAP regulated translation through EMT programme and the Wnt/β-catenin pathway. To prove that the decreased expression of EMT was regulated by YAP, we also detected EMT expression level after YAP overexpression in Hep-2 cells by RT-PCR and Western Blot. Compared with other groups, we found reduced expression of vimentin and increased expression of E-cadherin at both mRNA (Fig. 4a) and protein level (Fig. 4b) after transfection by YAP-shRNA. Additionally, we investigated protein expression of DKK1, β-catenin and GSK-3β in Hep-2 cells. The expression of DKK1 and β-catenin was lower that of the control group while GSK-3β was not significantly different. In both qRT-PCR and Western Blot assay, the expression of these key proteins in the YAP-NC and YAP-shRNA+YAP-cDNA groups was similar to control. These data strongly suggested that YAP regulated the EMT programme and Wnt/β-catenin pathway in Hep-2 cells.

### Downregulation of YAP inhibits Hep-2 tumourigenicity and metastasis in vivo

**Fig. 5:**
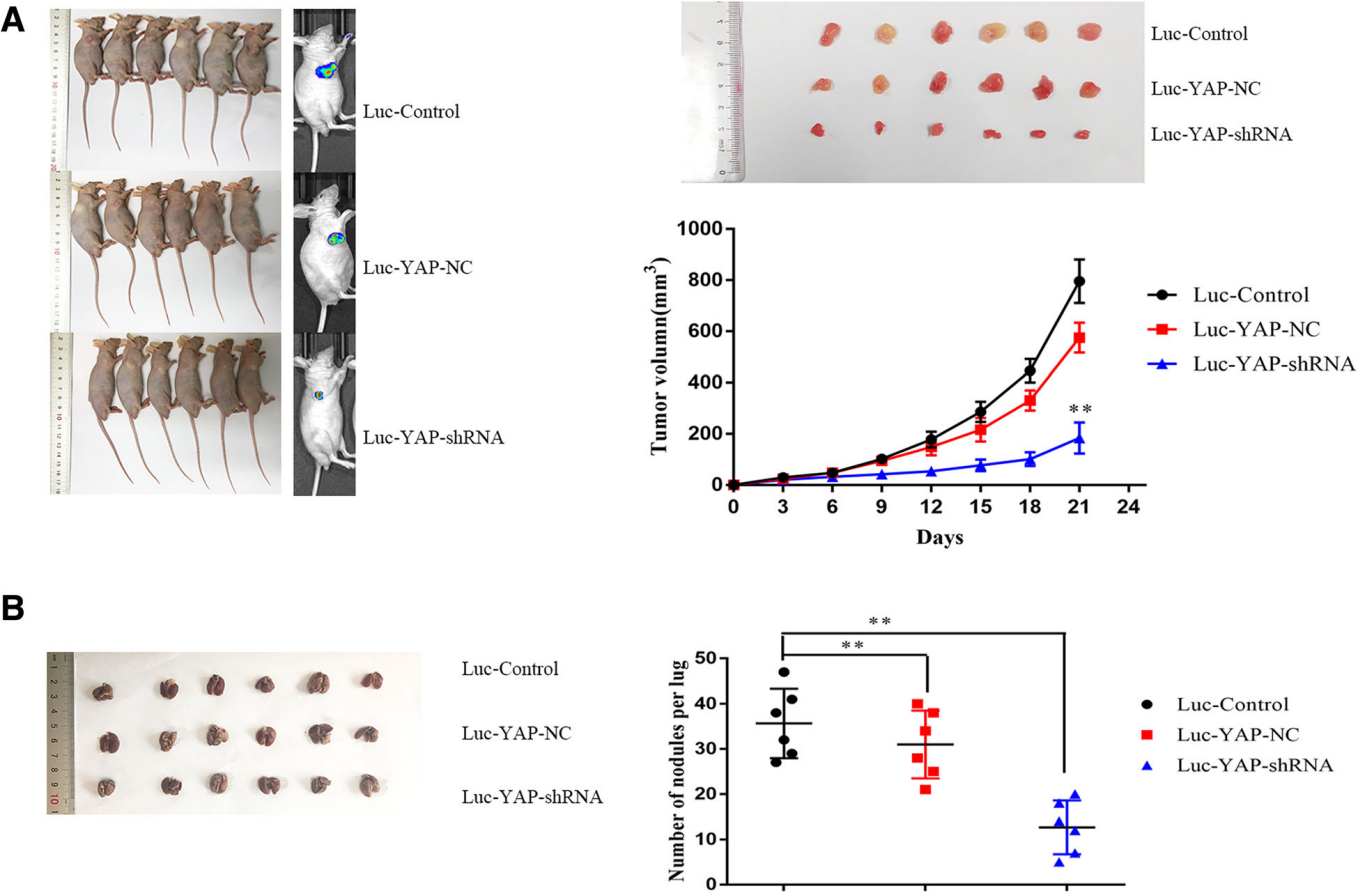
**a** Targeted silencing of YAP suppresses laryngeal cancer tumourigenicity and metastasis in vivo. The effects of silenced YAP on tumour suppression in vivo. Images of tumours (left panel) formed in nude mice injected subcutaneously with Hep-2 cells transfected with control, negative vector, and YAP-shRNA. Fluorescence images (right panel) of tumours captured on the IVIS system 21 days after subcutaneous injection. Cells of each group were stably transfected with luciferase. Tumours with YAP knockdown were smaller compared to the other groups. Tumour growth curves are plotted. **P < 0.01. **b** A pulmonary metastasis model (left panel) was established after 3 weeks of the indicated treatment. Images from the pulmonary metastasis model and the corresponding statistical analysis are shown. **P < 0.01

**Fig. 6:**
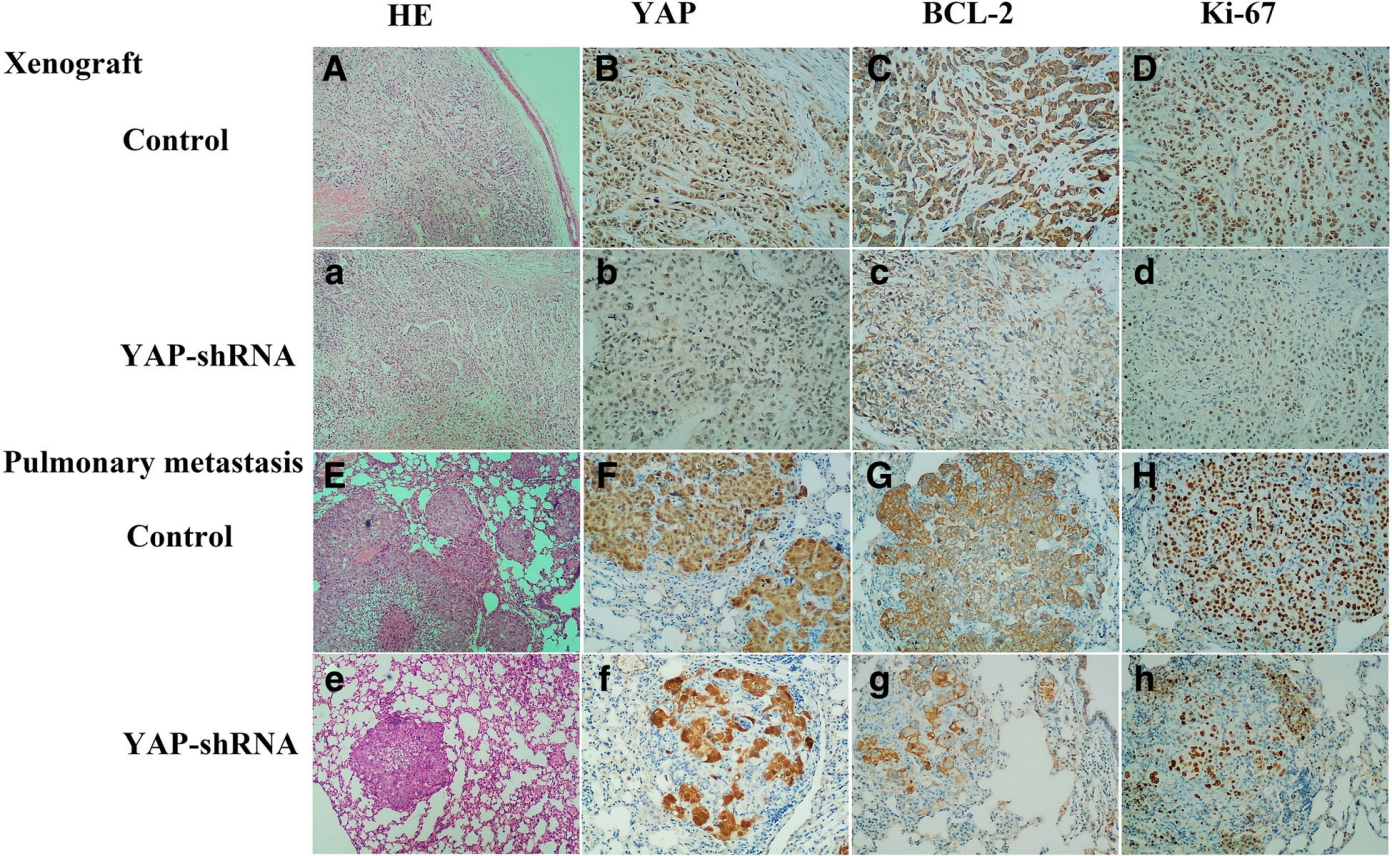
Immunohistochemistry staining for YAP, KI-67 and Bcl-2 protein in xenograft tumour model and pulmonary metastasis model. Cytoplasmic staining was considered to be positive for YAP, KI-67 and Bcl-2. **b**&**f** Higher YAP expression in both xenograft tumour and pulmonary models. **c**&**d** High YAP expression mainly in the nucleus of LSCC. **e**&**f** Low YAP expression in LSCC in both the cytoplasm and nucleus of tumour cells. **g**&**h** Low expression of YAP protein mainly in the nucleus of LSCC. [A, a, E, e] × 40; [B, b, C, c, D, d, F, f, G, g, H, h] × 100

To confirm the effect of YAP in Hep-2, we generated a luciferase-labelled Hep-2 cell line (Luc-control), a stable YAP downregulation Hep-2 cell line (Luc-YAP-shRNA) and a scrambled shRNA Hep-2 cell line (Luc-YAP-NC). We then designed a tumour model in nude mice. We injected different groups with cell suspensions subcutaneously and then measured each tumour volume every three days (Fig. 5a). After 21 days, the tumours were smaller than those in the control and YAP-NC groups, suggesting that the Luc-control and Luc-YAP-NC groups grew more aggressively. We also found similar results when using the IVIS spectrum in vivo imaging system. Then, we injected Hep-2 cells into the tail vein of nude mice and observed the development of lung tumours after three weeks. We detected fewer metastatic tumors in the pulmonary tissues of the downregulated group (Fig. 5b). Moreover, IHC assay (Fig. 6) exposed that the Luc-YAP-shRNA group exhibited less YAP, Bcl-2 and KI-67 staining, indicating that YAP-downregulated suppressed tumor growth of Hep-2 in vivo. These results demonstrated that targeted YAP downregulation repressed the tumour growth and metastasis of Hep-2 cells in vivo.

## Discussion

Laryngeal cancer is a common squamous-cell carcinoma malignancy that has the second highest incidence after nasopharyngeal cancer in otolaryngology [1]. In addition, the survival rate of patients with laryngeal cancer is low by reason of resistance to chemotherapy and radiotherapy and late metastases. Although the occurrence of cancer is a complex, multifactorial process, it is generally believed that in cancer gene activation, tumour suppressor gene inactivation and overexpression of anti-apoptotic genes are important causes of cancer. On the other side, the five-year post-treatment survival rate has remained poor [19] and the incidence of laryngeal cancer has increased [20] during the last 20 years. Thus, identifying new potential biomarkers and malignant indicators is of great urgency.

The Hippo signalling pathway is involved in the tumourigenicity of most tumours, while YAP is regarded as a vital downstream effector of the Hippo pathway. Activation of the Hippo signalling pathway leads to phosphorylation of YAP into the cytoplasm, resulting in inactivation of YAP gene transcription. The expression and nuclear localization of YAP was significantly elevated in numerous types of human cancers, such as liver cancer and lung cancer. Several recent reports have proposed that targeted YAP downregulation could restrain tumour cell proliferation and induce apoptosis, meaning that YAP might be a potential therapeutic target. Moreover, it is strongly related to histological differentiation, TNM stage, and poor prognosis. EMT can be affected by various signalling pathways, which is regarded as a critical programme in the early phase of the metastasis cascade [21]. Additionally, previous studies have indicated that EMT was required for epithelial cells to acquire malignant ability, while YAP is closely related to the EMT programme in CCA and breast cancer [22]. In our study, we found that abnormal activation of YAP caused proliferation in cancer cell, which also indicated that YAP could induce EMT in Hep-2 cells.

Wnt signalling is critical for development and homeostasis in metazoans with β-catenin being an important transcription activator, playing a pivotal role in tumour progression through the induction of epithelial-mesenchymal transition (EMT). Wnt signalling activation is important for tumourigenesis in various human cancers, some of which are with complicated signalling networks [23]. In fact, evidence from various experiments in vivo and *vitro* all confirmed that inappropriate activation of Wnt signalling mainly caused human neoplasia [24]. However, Wnt signalling pathway is regulated by the Hippo developmental pathway [17]. The extensive interrelationship of the Wnt and Hippo signalling pathways has emerged as an important tumourigenic signalling network in some cancer cells [25]. Moreover, YAP was recently identified as a novel Wnt regulator at the level of the destruction complex. At the same side, it was deem to be a transcriptional regulator of the Hippo pathway [26]. Increasing evidence [27] has confirmed that the close association about Wnt and Hippo signalling was emphasized by the role of YAP in the Wnt pathway.

The concept that YAP plays a crucial role in regulating the development of laryngeal cancer is further supported by our previous studies. First, targeted YAP downregulation significantly reduced the growth rate of Hep-2 and suppressed Hep-2 cell proliferation and colony formation while increasing the rate of apoptosis and inducing G2/M phase arrest in vitro. However, earlier study [28] shows that shRNA mediated downregulation of YAP induces G1 phase cell cycle arrest with apoptotic cell death. Another study [29] shows in BCPAP/KI cells siRNA mediated downregulation of YAP causes G0/G1 phase cell cycle arrest and autophasic cell death. The possible reason is that YAP-Hippo signaling pathway is regulated by different upstream pathways in cell cycle in each tumour.

Similarly, previous researches indicated that downregulating YAP suppressed proliferation and invasion of NSCLC and it might be a potential gene target for NSCLC treatment [30]. Second, in order to further investigate the potential therapeutic role of YAP in Hep-2 cells in vivo*,* we established subcutaneous and pulmonary metastasis tumour models based on our findings in vitro*.* According to our data, we revealed that downregulation of YAP prominently suppressed xenograft tumour growth and the formation of pulmonary metastatic tumors, implying that the silencing of YAP restrianed EMT-induced invasion and metastasis in Hep cells. Simultaneously, similar studies showed that YAP promotes ovarian cancer cell growth and tumourigenesis in vivo [31]. Third, we finally confirmed the role of YAP in EMT owing to elevated expression of the epithelial marker E-cadherin and reduced expression of the mesenchymal markers vimentin and β-catenin after YAP downregulation. As expected, there was an increase in mesenchymal markers and a reduction in epithelial markers due to overexpression of YAP, whereas silencing YAP had opposite effects. Other studies also found that knockdown of YAP decreased the protein level of vimentin and increased E-cadherin protein and mRNA levels in A549 cells [32]. Last, as we have shown in this study, the above data suggested a significant correlation between β-catenin mRNA and protein expression with YAP expression levels in Hep-2 cells, which verified that YAP regulated the proliferation and migration of Hep-2 via EMT and Wnt/β-catenin pathway. Moreover, high levels of biologically active YAP protein in Hep-2 cells were expected to stimulate proliferation of cancer cells and promote tumour progression. From the above, YAP may be regarded as a potential prognostic indicator and a potential anticancer molecular drug target for laryngeal cancer. Hence, the targeted-downregulation of YAP may be a logical approach for anti-tumour therapy in patients with laryngeal cancer. Nevertheless, its specific regulatory mechanism still needs further experimental exploration in the future.

## Conclusion

Taken together, our previous results demonstrated that YAP was obviously overexpressed in human laryngeal cancer tissues and its expression was involved in laryngeal cancer progression [7]. We first revealed that lentivirus-mediated YAP downregulation inhibited proliferation and tumourigenesis in human laryngeal carcinoma Hep-2 cells via EMT programme and Wnt/β-catenin pathway both in vitro and in vivo, indicating that disruption of YAP by lentivirus transduction may be a promising method for laryngeal cancer therapy. Therefore, we propose that YAP may be a potential prognostic biomarker and a credible approach for anti-tumour therapy for patients with laryngeal cancer.

## Data Availability

The data and materials used and analysed in the current study are available from the corresponding author on request.

## Abbreviations

CCK-8: Cell counting kit-8
cDNA: complementary deoxyribonucleic acid
DMEM: Dulbecco’s modified eagle’s medium
EMT: Epithelial-mesenchymal transition
GAPDH: Glyceraldehyde-3-phosphate dehydrogenase
IF: Immunofluorescence
IHC: Immunohistochemistry
NC: Negative control
NSCLC: Non-small cell lung cancer
PBS: Phosphate-buffered saline
PE: Phycoerythrin
qRT-PCR: quantitative real-time polymerase chain reaction
SD: Standard deviation
SEM: Standard error of the mean
shRNA: Short hairpin ribonucleic acid
TRITC: Tetramethylrhodamine
USA: United States of America
USTC: University of Science and Technology of China
WB: Western blot
YAP: Yes-associated protein
